# Altered volatile emission of pear trees under elevated atmospheric CO_2_ levels has no relevance to pear psyllid host choice

**DOI:** 10.1007/s11356-023-25260-w

**Published:** 2023-01-20

**Authors:** Jannicke Gallinger, Margit Rid-Moneta, Christine Becker, Annette Reineke, Jürgen Gross

**Affiliations:** 1 Institute for Plant Protection in Fruit Crops and Viticulture, Julius Kühn-Institut, Federal Research Institute for Cultivated Plants, Schwabenheimer Str. 101, 69221 Dossenheim, Germany; 2 Department of Ecology, Swedish University of Agricultural Sciences, Ulls Väg 16, 75007 Uppsala, Sweden; 3 Department of Crop Protection, Hochschule Geisenheim University, Von-Lade-Str. 1, 65366 Geisenheim, Germany

**Keywords:** Climate change, CO_2_ increase, Fruit production, Insect-plant interaction, Olfaction, Oviposition, Plant volatiles, Pear psyllid

## Abstract

The impact of climate change drivers on cultivated plants and pest insects has come into research focus. One of the most significant drivers is atmospheric carbon dioxide, which is converted into primary plant metabolites by photosynthesis. Increased atmospheric CO_2_ concentrations therefore affect plant chemistry. The chemical composition of non-volatile and volatile organic compounds of plants is used by insects to locate and identify suitable host plants for feeding and reproduction. We investigated whether elevated CO_2_ concentrations in the atmosphere affect the plant-pest interaction in a fruit crop of high economic importance in Europe. Therefore, potted pear trees were cultivated under specified CO_2_ conditions in a Free-Air Carbon dioxide Enrichment (FACE) facility at Geisenheim University in Germany for up to 14 weeks, beginning from bud swelling. We compared emitted volatiles from these pear trees cultivated for 7 and 14 weeks under two different CO_2_ levels (ambient: ca. 400 ppm and elevated: ca. 450 ppm CO_2_) and their impact on pest insect behavior. In total, we detected and analyzed 76 VOCs from pear trees. While we did not detect an overall change in VOC compositions, the relative release of single compounds changed in response to CO_2_ increase. Differences in VOC release were inconsistent over time (phenology stages) and between study years, indicating interactions with other climate parameters, such as temperature. Even though insect-plant interaction can rely on specific volatile compounds and specific mixtures of compounds, respectively, the changes of VOC patterns in our field study did not impact the host choice behavior of *C. pyri* females. In olfactometer trials, 64% and 60% of the females preferred the odor of pear trees cultivated under elevated CO_2_ for 7 and 14 weeks, respectively, over the odor from pear trees cultivated under ambient CO_2_. In binary-choice oviposition assays, *C. pyri* females laid most eggs on pears during April 2020; on average, 51.9 (± 51.3) eggs were laid on pears cultivated under eCO_2_ and 60.3 (± 48.7) eggs on aCO_2._

**Supplementary Information:**

The online version contains supplementary material available at 10.1007/s11356-023-25260-w.

## Introduction

Atmospheric carbon dioxide (CO_2_) concentrations are rising globally, mainly driven by economic growth and expansion of the human population (Feng et al. 2020). The greenhouse gas CO_2_ contributes to the global warming problem (IPCC 2014). Therefore, the reduction of anthropogenic CO_2_ emission was claimed as one of the most important goals by the Intergovernmental Panel on Climate Change (IPCC) in 2014 (IPCC 2014). While the increase of CO_2_ emission has slowed down in the European Union and the United States during the past decade, on a global level, the emission is still rising (Peters et al. 2020). Even though intensified production and cultivation practices of livestock and plants considerably contribute to the emission of greenhouse gases (Shakoor et al. 2021), agriculture itself is heavily affected by climate change, and its determinants are bearing new challenges for growers (Anderson et al. 2020). Changes of climate parameters and climate change drivers are impacting plant physiology as well as insect phenology, performance, and plant–insect interactions (Blande 2021; DeLucia et al. 2012; Forrest 2016; Jürgens and Bischoff 2017).

Regarding plant physiology, the global increase of carbon dioxide is of special interest, as plants utilize CO_2_ as carbon source to produce primary metabolites by photosynthesis. Therefore, higher CO_2_ concentrations have the potential to increase biomass production and crop yields and impact the nutritional quality of crops (Ainsworth and Long 2021; Dong et al. 2018; Wohlfahrt et al. 2018). In 2012, it was shown that growth under high CO_2_ concentrations increases photosynthetic rates in “Niitaka” pears (*Pyrus pyrifolia*) and resulted in higher soluble sugar contents in pear fruits (Han et al. 2012). A fertilizing effect of CO_2_ can increase the amount of primary metabolites, which are resources for production of secondary metabolites, such as volatile organic compounds (VOCs). Multifarious VOCs are released from plants. Characteristic plant VOC compositions are species, or even genotype and developmental stage specific, as well as inducible by biotic and abiotic stresses (Gallinger et al. 2020; Rid et al. 2019; Hickman et al. 2021). Thus, VOCs provide information regarding the identity and condition of the releasing plant to other organisms in their environment (Bouwmeester et al. 2019). Regarding the effect of CO_2_ on plant VOCs, it is shown that the exposition of oilseed rape plants to elevated CO_2_ concentrations (720 µl l^−1^) increased the constitutive emission of several terpenoids (Himanen et al. 2009). Such plant-derived VOCs are important for the intraspecific communication of plants. For insects, olfactory, besides visual, cues are important for the orientated movement (searching behavior) over distance (Deletre et al. 2016; Schoonhoven et al. 2005). Therefore, a change in the VOC composition emitted by plants under increased carbon dioxide concentrations can impact insect behavior (Chen et al. 2019).

Only a small number of studies have been carried out under field conditions because sophisticated facilities are needed to manipulate CO_2_ levels in open-field situations (Ainsworth and Long 2005, 2021). Especially, studies with slow-growing perennial plants are rare. To date, there is no study that has investigated the impact of CO_2_ concentrations on the interaction of pear trees and one of their most threatening pests, pear psyllids.

Psyllids or jumping plant lice belong to the order of Hemiptera and feed with their piercing-sucking mouth parts on the phloem tissue of their host plants. Psyllids are serious pests of several cultivated plants. Species in the genus *Cacopsylla* are known to be harmful to fruit trees, and the pear psyllid *Cacopsylla pyri* is a major pest in pear-growing regions all over Europe (Garcia-Chapa et al. 2005). *C. pyri* is damaging fruit trees directly and indirectly. Pear trees can suffer from massive psyllid infestations, due to direct feeding damage. In the event of mass occurrence, enormous amounts of honeydew, especially from nymphs, are excreted, serving as a medium for sooty mold fungus (Burckhardt 1994). Additionally, *C. pyri* is a vector of the phytopathogen “*Candidatus* Phytoplasma pyri”, a specialized cell wall–less bacterium colonizing the insect vector and the phloem tissue of pear trees (Carraro et al. 1998). While feeding on the phloem sap of host trees, psyllids can transmit “*Ca.* P. pyri” from plant to plant (Jarausch et al. 2019). This bacterium induces the so-called pear decline, a severe disease in pear-growing. Hence, *C. pyri* is of high economic relevance.

A number of studies on different species demonstrated that psyllids perceive plant volatiles and evaluated the role of plant chemical cues for host finding by psyllids (Alquézar et al. 2017; Coutinho-Abreu et al. 2014; Gallinger et al. 2020; George et al. 2016; Gross et al. 2019b; Kristoffersen et al. 2008; Mayer et al. 2008a, 2008b; Nehlin et al. 1994; Rid et al. 2016; Soroker et al. 2004; Yuvaraj et al. 2013). The detection of suitable host plants is crucial for survival and reproductive success of herbivorous insects. Therefore, we hypothesize that changes in volatile emission of host plants can impact the host preference of pear psyllids, which may impact their distribution and the dispersion of “*Ca.* P. pyri” in the future. Therefore, we firstly investigated whether increased CO_2_ concentrations affect the emission of VOCs from pear trees. This was realized under field conditions in a Free-Air Carbon dioxide Enrichment (FACE) facility where potted pear trees were placed under different CO_2_ levels before bud break. Further, we evaluated how changes in the VOC profiles impact the host choice of *C. pyri* females and thus potentially alter psyllid pest pressure in pear orchards under future conditions.

## Materials and methods

### Plants and insects

**Fig. 1:**
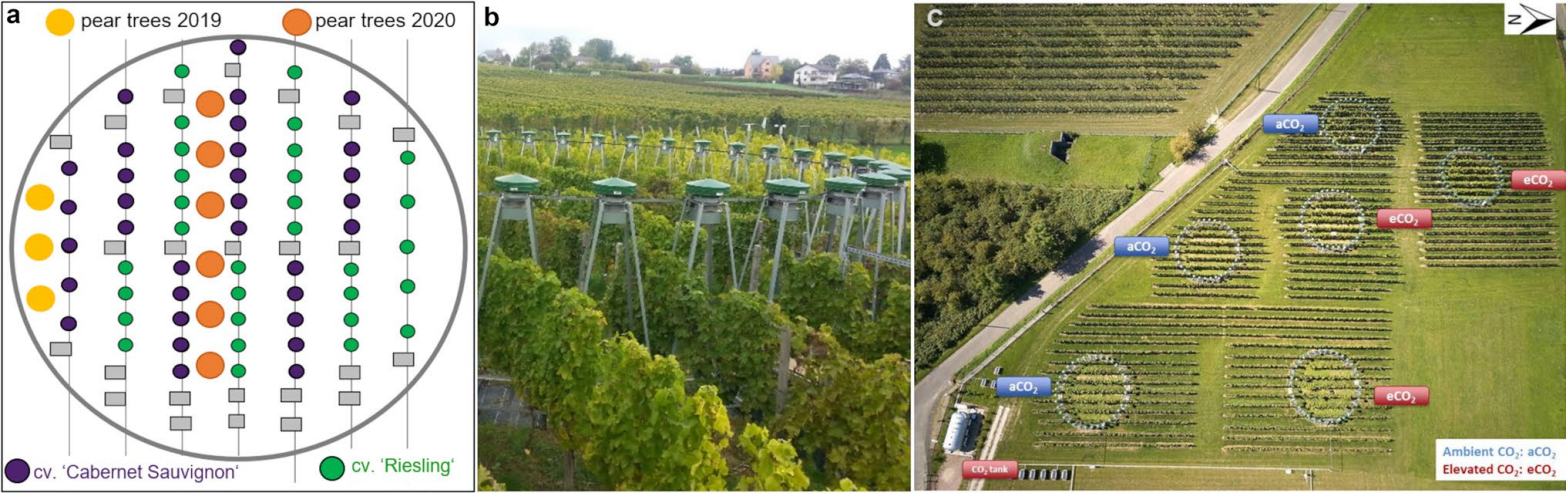
**a** Position of potted pear trees 2019 and 2020 in the inter-rows of the VineyardFACE facility at Geisenheim University, Germany. **b** Close-up of a FACE ring, consisting of 36 jets mounted at a height of 2.5 m equipped with fans for CO_2_ distribution. **c** Overview of the VineyardFACE experimental site, with three FACE rings assigned to the two CO_2_ concentrations, ambient CO_2_ (aCO_2_ ~ 400 ppm) and elevated CO_2_ (eCO_2_ ~ 450 ppm), and the associated CO_2_ tank

Two-year-old *Pyrus communis* cv. Williams Christ trees (rootstock: quince BA 29) obtained from a nursery (Jäger, Ladenburg, Germany) were used for the experiments. In both years (2019 and 2020), trees were potted in 25-L pots and pruned in February. Afterwards (2019: February 25, 2019, and 2020: March 2, 2020), the potted pear trees were placed in the inter-rows of the VineyardFACE facility at Geisenheim University, Germany, for a total period of 13 weeks in 2019 and 14 weeks in 2020. The FACE facility consisted of six rings; in three rings, an elevated concentration of CO_2_ (eCO_2_, approx. 450 ppm) was established, whereas in the other three rings, the ambient CO_2_ level (aCO_2_, approx. 400 ppm) was not modified (Fig. 1c). For a detailed description of the Geisenheim VineyardFACE facility, see publications by Reineke and Selim (2019) and Wohlfahrt et al. (2018). In 2019, three pear trees were placed at one edge of each ring, resulting in nine trees each for ambient and elevated CO_2_ concentration (Fig. 1a–c). In 2020, the number of pear trees per ring was increased to six, and the trees were positioned in the middle of each ring, resulting in 18 trees for each CO_2_ concentration (Fig. 1a). In 2022, half of the trees (three trees per ring) were removed from the FACE facility after 7 weeks and used for behavioral experiments with pear psyllids.

All pear trees were fertilized once at the beginning of the growing season with approx. 20-g slow-release fertilizer (Triabon 16–8-12 (+ 4 + TE), COMPO EXPERT GmbH, Münster, Germany). All plants were watered whenever necessary. Phenological growth stages of pear trees at the different sampling dates were classified following the BBCH identification key for pome fruits by Meier (2018): principal growth stage 3, shoot development; and secondary stages 1–9, shoots about 10–90% of final length.

Additional *P. communis* (cv. Williams Christ on Kirchensaller Mostbirne) were grown for the insect rearing and the no-choice oviposition assays, in an insect save screen house under ambient climatic conditions. Pear trees were fertilized with ~ 500-mL Triabon (COMPO Expert GmbH, Münster, Germany, 2 g/L) once (in March).

Adult *C. pyri* (summer form) were caught from *P. communis* trees at the experimental field of the Julius Kühn-Institut (JKI) in Dossenheim, Germany (49°27′00.0″N, 8°38′24.0″E), by beating tray method. A square beat sheet was placed under a branch of a pear tree; the branch was hit with a foam plastic-covered stick, causing the psyllids to fall on the sheet (Weintraub and Gross 2013). All psyllids were collected from the sheet. Species and sex was determined under a stereo microscope (Stemi 2000, Zeiss, Oberkochen, Germany). Captured *C. pyri* adults were either directly used for experiments (olfactometer experiments within 24 h) or kept in a climate chamber at 20 °C (photophase) and 16 °C (scotophase) (L16:D8) until they were used in oviposition experiments. Therefore, collected C*. pyri* adults were kept on *P.* *communis* (cv. Williams Christ) plants in screen cages (BugDorm, MegaView Science Co, Taiwan, 47.5 × 47.5 × 93 cm).

### Volatile collection

Volatile emissions were sampled from potted *P. communis* trees in a non-destructive way in the field (FACE facility) at two phenological growth stages (shoot development) per year (2019: BBCH 32–33, May 7, 2019, and BBCH 38–39, June 4, 2019; 2020: BBCH 32–33, April 21, 2020, and BBCH 38–39, June 2, 2020). In 2019, the volatiles from nine trees per CO_2_ condition were sampled at both sampling dates. In 2020, the volatiles from 18 pear trees per CO_2_ condition were sampled at the first sampling date (BBCH 32–33, April 21, 2020); afterwards, half of the trees were removed from the FACE facility and used for behavioral studies, resulting in a total number of nine pear trees per CO_2_ condition that were sampled at the second BBCH stage (BBCH 38–39, June 2, 2020). Samples were taken with a mobile 6-channel headspace sampling device composed of six mass flow controllers (M + W Instruments GmbH, Leonhardsbuch, Germany) each controlling one vacuum pump (KNF Neuberger GmbH, Freiburg, Germany) as described by Rid et al. (2016). One single branch of each tree was carefully wrapped in polyethylene terephthalate oven bags (Toppits, Melitta, Minden, Germany). Clean air filter cartridges (ICAF 2X6, Sigma Scientific, Micanopy, USA) were used to filter the ambient air. The clean air was pumped through the oven bags with a flow of 1000 mL/min. Volatiles were trapped on stainless steel, prepacked sample tubes containing 200-mg Tenax TA35/60 sorbent (Markes, Neu-Isenburg, Germany). A total volume of 30 L was pumped over the matrix. Samples were analyzed by GC–MS analysis within 1 week.

### GC–MS analysis

Volatile samples were analyzed with an automated thermal desorber (TurboMatrix™ ATD 650, PerkinElmer, Rodgau, Germany) connected to a gas chromatograph (Clarus R 680, PerkinElmer) coupled to a Perkin Elmer quadrupole inert mass selective detector (ATD-GC–MS). Sample tubes were desorbed for 10 min at 250 °C. A Tenax TA–filled cold trap was held at − 20 °C through the desorption followed by heating at a rate of 99 K/s to 250 °C and 1 min desorption time. A nonpolar Elite-5MS (Crossbond 5% diphenyl/95% dimethyl polysiloxane, PerkinElmer) capillary column (30 × 0.25-mm id × 0.25-μm film thickness) was used for GC separation of volatile compounds. Helium was used as carrier gas (Helium 6.0, Linde, Munich, Germany) with a column head pressure of 130 kPa. The GC temperature program was as follows: Initial oven temperature of 40 °C was held for 1 min, increased at a rate of 5 K/min to 180 °C, followed by a rate of 20 K/min to the final temperature of 280 °C, and held for 6 min. The GC inlet line temperature was 250 °C, and the ion source temperature was 180 °C. The quadrupole mass detector was operated in the electron impact (EI) mode at 70 eV. All data were obtained by collecting the full-scan mass spectra within the range of 35–350 m/z, resulting in the following number of chromatograms per year and BBCH stage used in statistical analysis, in 2019: *n*_aCO2BBCH32–33_ = 8, *n*_eCO2BBCH32–33_ = 7, *n*_aCO2BBCH38–39_ = 9, and *n*_eCO2BBCH38–39_ = 9, and in 2020: *n*_aCO2BBCH32–33_ = 18, *n*_eCO2BBCH32–33_ = 18, *n*_aCO2BBCH38–39_ = 9, and *n*_eCO2BBCH38–39_ = 9.

### Identification and quantification with AMDIS

Chromatograms of volatile samples were analyzed using “Automated Mass spectral Deconvolution and Identification System” (AMDIS, V. 2.71; National Institute of Standards and Technology NIST, Boulder, CO). For the identification, the ion fragmentation patterns and retention indices of detected compounds were compared with standard compounds according to Gross et al. (2019a). Not identified compounds were annotated as unknowns. For quantification, the peak areas were integrated after deconvolution. Identification criteria were applied as follows: match factor had to be ≥ 80% and the relative retention index deviation ≤ 5% from reference value. The settings for deconvolution were component width, 32; adjacent peak subtraction, one; resolution, low; sensitivity, medium; shape requirements, low; level, very strong; maximum penalty, 20; and “no RI in library” 20. Components with a signal to noise ratio < 50 were excluded from the analysis.

### Olfactometer assays

The preference of *C. pyri* females for odors of *P.* *communis* trees cultivated under different CO_2_ levels was evaluated in a dynamic Y-shaped olfactometer, after 7 weeks (BBCH 32–33) and 14 weeks (BBCH 38–39) of cultivation in the FACE facility. The Y-shaped glass tube was mounted on a board in an angle of 40° from the horizontal plane*.* The entrance arm length of the tube was 12.5 cm, the test arm length 8 cm, and the inner diameter 1 cm; test arms had an angle of 75°. A light source (LED-Lupenleuchte, Purelite, UK) was mounted 45 cm above the middle of the olfactometer, resulting to approx. 280 lx at the experimental area. All experiments were conducted between 09:00 a.m. and 6:00 p.m. at room temperature (20–26 °C and 30–35% RH). One potted pear tree per CO_2_ condition and growth stage was transferred from the FACE facility in Geisenheim to the JKI in Dossenheim. The trees were used for olfactometer assays 2 days in the following. One twig of each tree was carefully wrapped in oven plastic bags (Toppits, Melitta, Minden, Germany, 31 × 50 cm) and connected to the test arms. Charcoal-filtered and humidified air was pumped through the plastic bags into the test arms. The air flow of each test arm was adjusted to 75 mL/min (with a max. difference of 1 mL/min) by plastic valves and controlled by a flowmeter (MASS-STREAM, M + W Instruments, Allershausen, Germany) to ensure an equal airstream in both arms. The odors of *P. communis* (cv. Williams Christ on BA29) trees cultivated under elevated (approx. 450 ppm) or ambient (approx. 400 ppm) CO_2_ levels in the FACE facility were offered simultaneously or against clean air to *C. pyri* females. Psyllids were collected from the experimental field at the JKI and kept in reaction vials in the dark prior to olfactometer tests. Each psyllid was released from the reaction tube at the base of the entrance arm. To prevent side effects, the olfactometer was rotated 180° after five replicates. The number of females that entered one of the test arms (1 cm) and stayed for at least 30 s was counted. Psyllids that did not reach one of the test arms within 10 min were recorded as “no choice.” Olfactometers and accessories were cleaned with ethanol (70%) and heated at 230 °C (except plastic valves: 60 °C) for 2 h. Previous studies showed changes in psyllid behavior due to phytoplasma infections (Mayer et al. 2008a); therefore, infection status of *C. pyri* females was determined. Females from olfactometer assays were frozen at − 20 °C and tested for phytoplasma infection by DNA extraction followed by PCR and gel electrophoresis. Choices of females, which were infected with phytoplasmas, were excluded from the analysis. In total, the behavior of 46 to 52 females per comparison was observed.

### Oviposition assays

To investigate the oviposition behavior of *C. pyri*, we conducted (“No-choice” section) no-choice experiments and (“Binary-choice” section) binary-choice experiments under the following conditions.

#### No-choice

No-choice oviposition assays were conducted to investigate a direct and short-term effect of CO_2_ on the oviposition preference of *C. pyri* females. Therefore, the assays were conducted in the presence of ambient (400 ppm) or strongly elevated (750 ppm) CO_2_ concentrations in a climate cabinet (Rumed P 1060, Rubarth Apparate GmbH, Laatzen, Germany) under controlled climate conditions at 20 °C during photophase and 16 °C during scotophase (L16:D8) and 60 ± 5% relative humidity. In total, thirty *C. pyri* females were placed on leaves of the additional planted *P. communis* (cv. Williams Christ on Kirchensaller Mostbirne) plants from the screen house per CO_2_ concentration. Each female was individually caged on one leaf with a gauze bag (10 × 12 cm). Two trees were used for each CO_2_ condition, and 15 females were caged at each tree (*n*_aCO2_ = 30, *n*_eCO2_ = 30). After 96 h, the leaves with the psyllids were detached from the pear trees and stored at − 20 °C until the number of eggs was counted under a stereomicroscope (Stemi 508, Carl Zeiss AG, Oberkochen, Germany). Oviposition assay was conducted in August 2019 with females from the third generation of reared *C. pyri* (as described in the”Plants and insects” section).

#### Binary-choice

Binary-choice assays were conducted with *P. communis* (cv. Williams Christ on BA29) trees grown under ambient (approx. 400 ppm) or elevated (approx. 450 ppm) CO_2_ levels. Therefore, eight of the potted pear trees per CO_2_ level were removed from the Geisenheim VineyardFACE facility (16 in total). The oviposition assays were conducted immediately after removing the trees from the facility in an insect save screen house at Geisenheim University. One intact leaf each from a tree grown under ambient and elevated CO_2_ concentrations was offered simultaneously, by inserting one intact leaf of each tree from opposite sides into a gauze sleeve (20 × 30 cm). In 2019 a single female (n_BBCH38-39_ = 47) and in 2020 one pair of *C. pyri* adults (one of each sex) was introduced in each bag (n_BBCH32–33_ = 49, n_BBCH38–39_ = 47). In both years, psyllids were allowed to oviposit for 5 days (120 h). Following this, the leaves were removed from the plants and frozen at − 20 °C. The number of eggs laid on each leaf was counted under a stereomicroscope (Stemi 508, Carl Zeiss AG, Oberkochen, Germany).

### Statistical analysis

Statistical analysis was done in R version 4.0.3. (R Core Team 2020).

Volatile profiles: “vegan” package was used for multivariate analysis of volatile profiles from *P.* *communis* trees (Oksanen et al. 2020). A Bray–Curtis dissimilarity matrix was calculated from the compositional data set of volatiles with the *vegdist* function. Non-metric multidimensional scaling (NMDS) plots were used to visualize Bray–Curtis dissimilarities with the *metaMDS* function. Scaling was standardized by Wisconsin double standardization. Permutational multivariate analysis of variance (PERMANOVA) was used to analyze differences in volatile compositions between the CO_2_ treatments and sampling dates (BBCH stages), with the *adonis* function (*N* permutations = 10.000). Additionally, the dispersion of groups was tested for multivariate homogeneity. The relative amount of single volatiles in the profiles was compared between pears grown under aCO_2_ and eCO_2_ conditions with a nonparametric Mann–Whitney *U*-test.

Olfactometer assays: Preference for a host plant volatile profile was evaluated with the binominal test.

Oviposition: In binary-choice test, replicates where no eggs were laid were excluded from the analysis. In no-choice tests, zeros were not excluded, because they could represent avoidance behavior. Zero-inflated negative binomial (ZINB) models were fitted to evaluate the difference in number of eggs laid on leaves from pear trees grown under aCO_2_ and eCO_2_ conditions in the binary-choice experiment. The use of zero-inflated models provides the possibility to estimate the effect of covariates on the probability to encounter a zero in the count data. ZINB models were generated with the *zeroinfl* function from “pscl” package (Zeileis et al. 2008). The log-transformed total number of eggs per female was used as offset. We used AIC model selection to compare models that include or not include the combination of trees as factors. Integration of the tree combination as model factor did not increase the model fit and was therefore not included in the models.

Differences in oviposition choice depending on the growth condition (CO_2_ concentration) of the pear trees were calculated using estimated marginal means and 95% confidence intervals with the *emmeans* function from “emmeans” package (Lenth 2021).

## Results

### Volatile emission

In total, 76 components (Table S1) were annotated from the chromatograms and compared between the CO_2_ treatments at two growth stages.

**Fig. 2:**
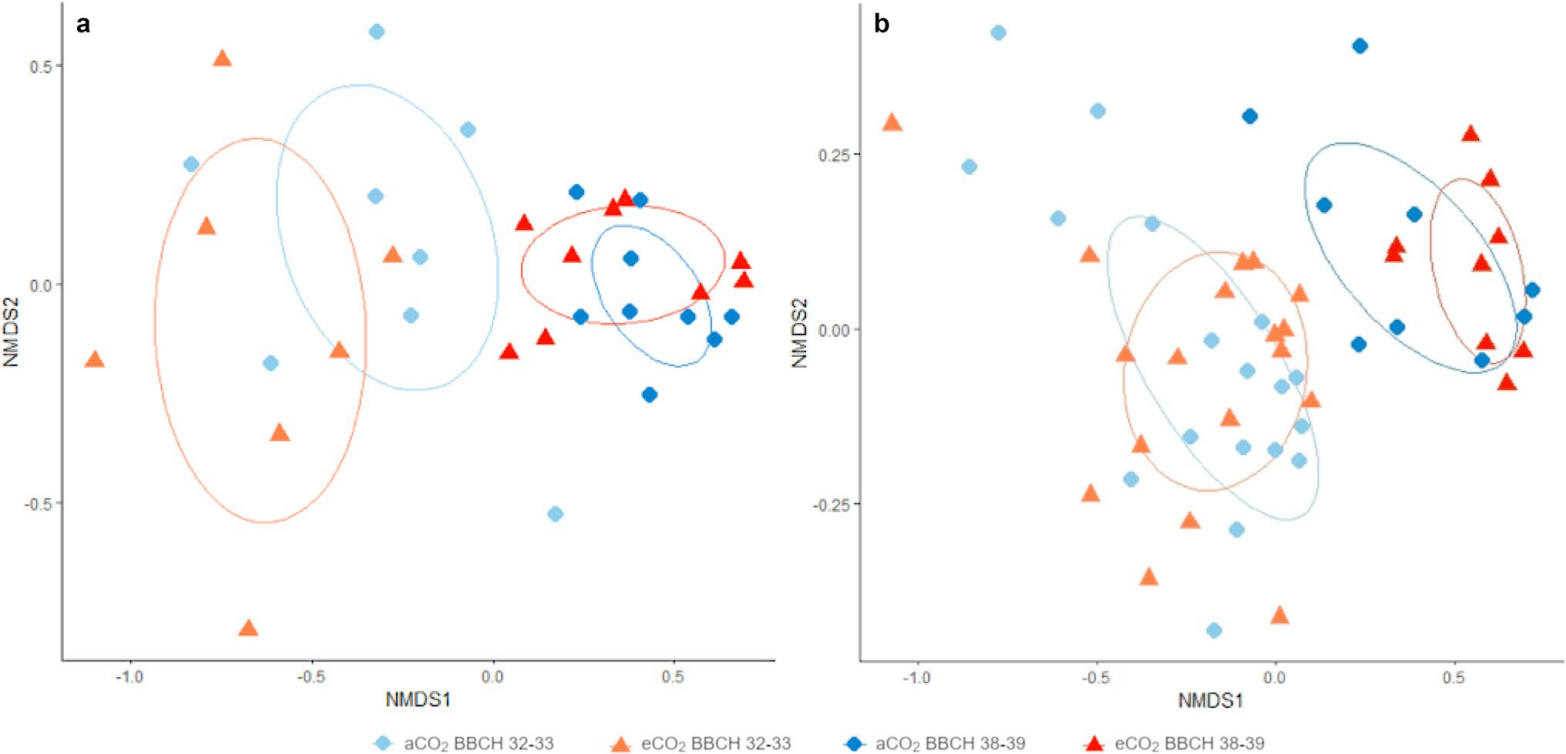
Non-metric multidimensional scaling (NMDS) plot based on Bray–Curtis dissimilarities calculated from proportional volatiles compositions of *Pyrus communis* trees grown under ambient (blue circles) or elevated (red triangles) CO_2_ conditions at two different phenological stages in the FACE system. **a** 2019 (2d-stress: 0.111; *n*_aCO2BBCH32–33_ = 8, *n*_eCO2BBCH32–33_ = 7, *n*_aCO2BBCH38–39_ = 9, *n*_eCO2BBCH38–39_ = 9) and **b** 2020 (2d-stress: 0.107; *n*_aCO2BBCH32–33_ = 18, *n*_eCO2BBCH32–33_ = 18, *n*_aCO2BBCH38–39_ = 9, *n*_eCO2BBCH38–39_ = 9)

In both years, the volatile composition emitted from pear trees at the two investigated phenological stages differed significantly (Fig. 2, (a) 2019: perMANOVA, *F* = 9.8859, *P* = 0.0002, *N* = 33; (b) 2020: perMANOVA, *F* = 13.98, *P* = 0.00009, *N* = 54). The CO_2_ concentration as well as the interaction between the phenological stage and the CO_2_ concentration had no significant effect on the discrimination between the volatile profiles (Fig. 2, (a) 2019: perMANOVA, *F* = 1.04, *P* = 0.361, *N* = 33; (b) 2020: perMANOVA, *F* = 0.96, *P* = 0.430, *N* = 54).

**Fig. 3:**
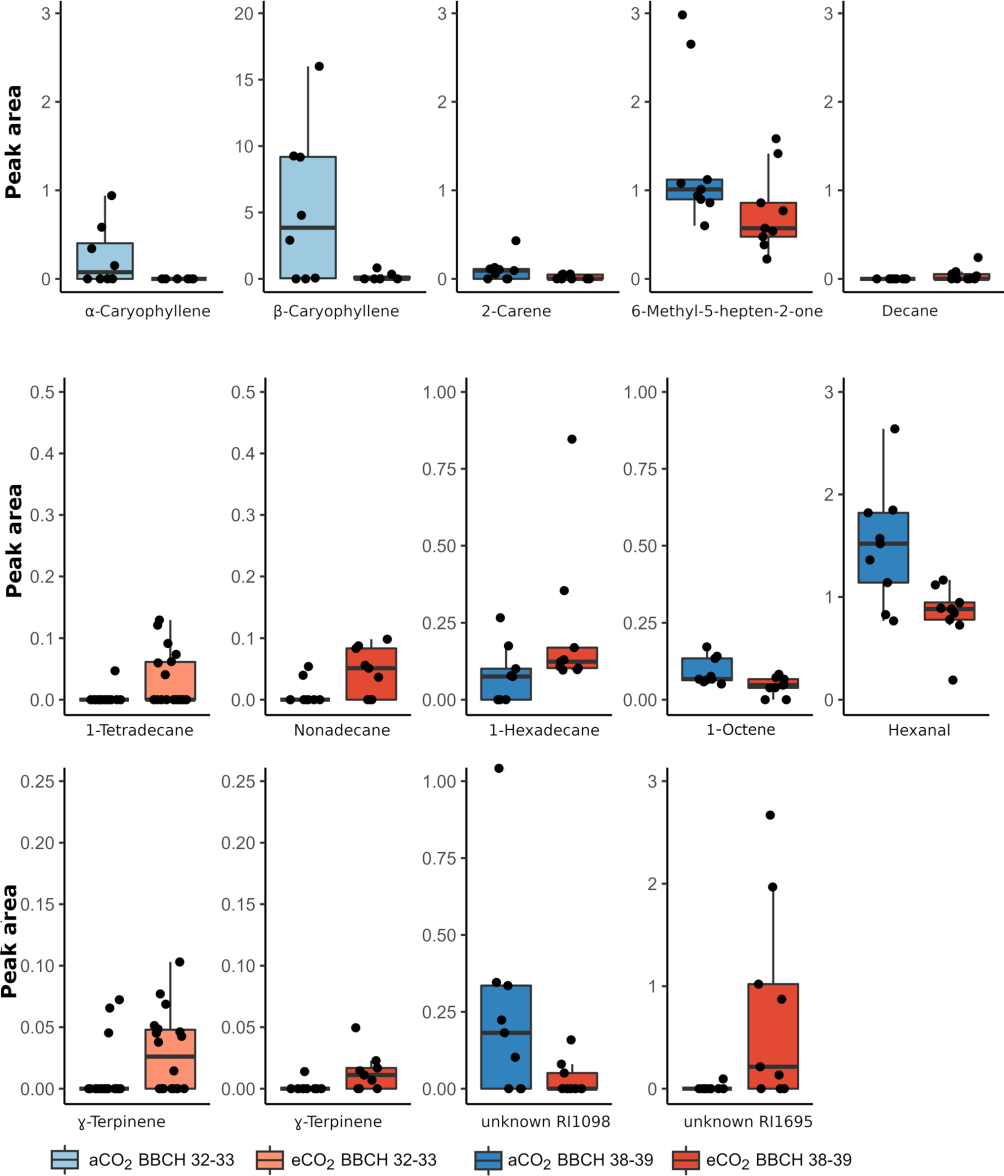
Box-Whisker plots with jittered raw values of relative amount of single volatile compounds from *Pyrus communis* trees grown under ambient (blue) or elevated (red) CO_2_ conditions in the FACE system. **a** 2019 at BBCH 32–33 (*n*_aCO2_ = 8, *n*_aCO2_ = 7) and BBCH 38–39 (*n*_aCO2_ = 9, *n*_aCO2_ = 9), **b** 2020 at BBCH 32–33 (*n*_aCO2_ = 18, *n*_aCO2_ = 18) and BBCH 38–39 (*n*_aCO2_ = 9, *n*_aCO2_ = 9). Medians are shown as lines, and whiskers extend to 1.5 times of the interquartile ranges

Single volatiles were emitted in significantly different amounts from pear trees under the two CO_2_ concentrations. In 2019, pear trees at the early phenological stage (BBCH 32–33) released significantly less α- and β-caryophyllene (Fig. 3a, Mann–Whitney *U*, α-caryophyllene: *Z* = 2.08, *P* = 0.038, *N* = 15; β-caryophyllene: *Z* = 2.07, *P* = 0.038, *N* = 15) under eCO_2_ than aCO_2_ condition. Later, in the season (BBCH 38–39), the release of caryophyllene did not differ (Mann–Whitney *U*, α-caryophyllene: *Z* = 1.29, *P* = 0.199, *N* = 18; β-caryophyllene: *Z* = 0.22, *P* = 0.825, *N* = 18) between CO_2_ conditions. Pear trees under eCO_2_ conditions released significantly lower amounts of 2-carene (Fig. 3a, Mann–Whitney *U*, *Z* = 2.12, *P* = 0.034, *N* = 18) and 6-methyl-5-hepten-2-one (Fig. 3a, Mann–Whitney *U*, *Z* = 2.16, *P* = 0.031, *N* = 18) than under aCO_2_ conditions, whereas the emission of decane (Fig. 3a, Mann–Whitney *U*, *Z* =  − 2.18, *P* = 0.029, *N* = 18) was increased under eCO_2_ compared to aCO_2_ conditions.

In 2020, pear trees at early growth stage (BBCH32–33) released significantly higher amounts of 1-tetradecane (Fig. 3b, Mann–Whitney *U*, *Z* =  − 2.46, *P* = 0.014, *N* = 36) and γ-terpinene (Fig. 3b, Mann–Whitney *U*, *Z* =  − 2.17, *P* = 0.03, *N* = 36) when grown under eCO_2_ than under aCO_2_ condition. Later, in the season (BBCH38–39), elevated CO_2_ levels lead to increased amounts of nonadecane (Fig. 3b, Mann–Whitney *U*, *Z* =  − 2.03, *P* = 0.042, *N* = 18), 1-hexadecene (Fig. 3b, Mann–Whitney *U*, *Z* =  − 2.17, *P* = 0.03, *N* = 18), γ-terpinene (Fig. 2b, Mann–Whitney *U*, *Z* =  − 2.36, *P* = 0.018, *N* = 18), and one unidentified component RI1695 (Fig. 3b, Mann–Whitney *U*, *Z* =  − 2.56, *P* = 0.010, *N* = 18) in the overall composition. The release of 1-octene (Fig. 3b, Mann–Whitney *U*, *Z* = 2.25, *P* = 0.024, *N* = 18), hexanal (Fig. 3b, Mann–Whitney *U*, *Z* = 2.34, *P* = 0.019, *N* = 18), and one unidentified component RI1098 (Fig. 3b, Mann–Whitney *U*, *Z* = 2.03, *P* = 0.043, *N* = 18) was reduced in odor profiles of pear trees cultivated under elevated compared to ambient CO_2_ concentration.

### Olfactometer assays

**Fig. 4:**
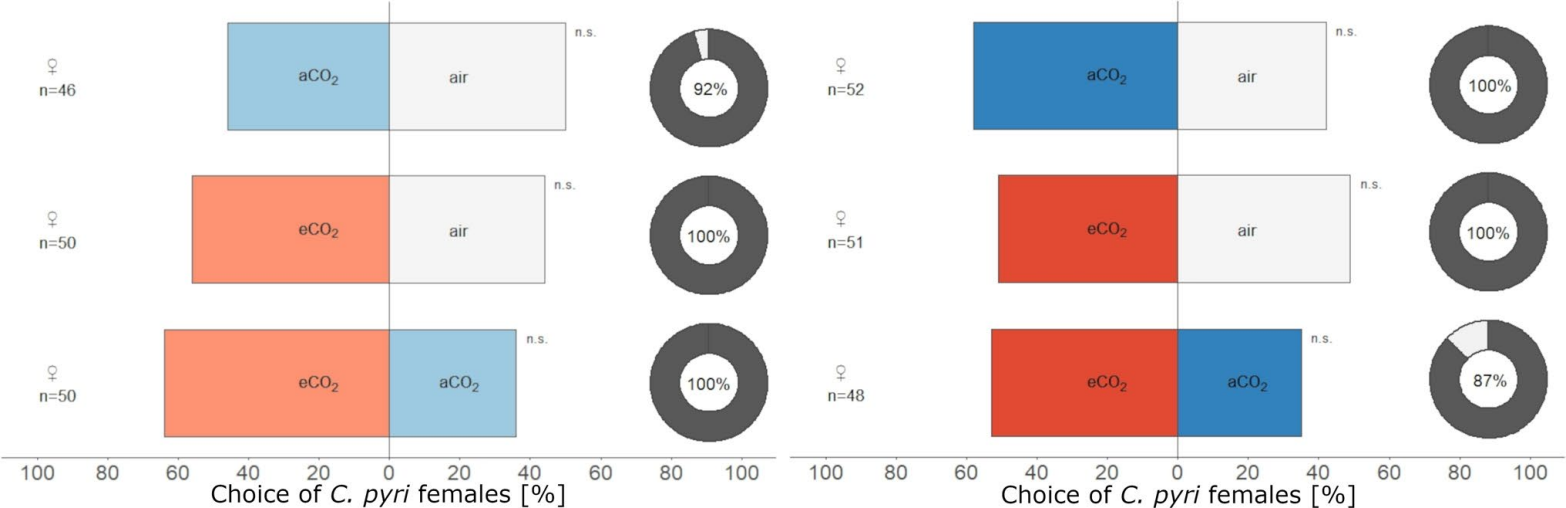
Choice of *C. pyri* females in a Y-tube olfactometer for odor profiles of *Pyrus communis* trees grown under ambient (approx. 400 ppm, blue) or elevated (approx. 450 ppm, red) CO_2_ levels at **a** BBCH 32–33 and **b** BBCH 38–39 in 2020. Percentage of psyllids that made a choice (dark gray) and that did not (light gray) is presented as pie chart on the right (n.s. = not significant, binominal test)

*Cacopsylla pyri* females did not significantly prefer odor blends from *P. communis* plants over clean air in olfactometer assays at BBCH 32–33 (Fig. 4a, binominal test, aCO_2_: *P* = 0.883, *N* = 46; eCO_2_: *P* = 0.48, *N* = 50) nor at BBCH 38–39 (Fig. 4b, binominal test, aCO_2_: *P* = 0.332, *N* = 52; eCO_2_: *P* = 1, *N* = 51). Additionally, females did not show a preference for odors from *P. communis* plants grown under elevated or ambient CO_2_ levels in the FACE environment (Fig. 4, (a) BBCH 32–33: binominal test, *P* = 0.065, *N* = 50; (b) BBCH 38–39: binominal test, *P* = 0.193, *N* = 48). When offered simultaneously, 64% of female *C. pyri* walked in the direction of the odor from eCO_2_ pears at BBCH 32–33 (Fig. 4a) and 60% at BBCH 38–39 (Fig. 4b). General motivation of females was high and ranged from 87 to 100% (Fig. 4).

### Oviposition

*Cacopsylla pyri* females did not show significant oviposition preference for *P. communis* trees grown under ambient or elevated CO_2_ levels in no-choice and binary-choice bioassays (Table 1). In no-choice bioassay, *C. pyri* females laid on average 18.3 (± 29.9) eggs on pear leaves from ambient CO_2_ and 24.4 (± 30.7) eggs from elevated CO_2_ grown trees. Given a choice between two leaves from trees grown under ambient or elevated CO_2_ concentrations, *C. pyri* females laid on average 60.3 (± 48.7) eggs on aCO_2_ and 51.9 (± 51.3) eggs on eCO_2_ grown pear leaves at BBCH 32–33; 21.7 (± 30.9) and 21.7 (± 24.3) eggs on aCO_2_ and eCO_2_ grown leaves, respectively, at BBCH 38–39 in 2019; and 21.0 (± 24.4) and 16.1 (± 22.4) eggs on aCO_2_ and eCO_2_ grown leaves, respectively, at BBCH 38–39 in 2020. In general, *C. pyri* females laid twice as many eggs in experiments with pear trees in early growth stage (BBCH 32–33) in April 2020 compared to binary-choice experiments later in the season (BBCH 38–39; Table 1).

**Table 1:** Model estimations and statistics and total number of eggs laid by *C. pyri* females in no-choice and binary-choice experiments on pear trees grown under ambient or elevated CO_2_ concentrations. Conducted in 2019 and 2020 at different phenological growth stages

Choice	Year/BBCH	Estimated nr. of eggs / leaf (CI)		Model stats		Total number of eggs	
		Ambient CO_2_	Elevated CO_2_	*Z*_eggs_ *Z*_0_	*P*_eggs_ *P*_0_	aCO_2_	eCO_2_
No *	2019/38–39	41.7 (25.2–69.0)	38.0 (25.0–57.7)	− 0.282 − 1.545	0.778 0.122	550	733
Binary**	2019/38–39	40.2 (32.3–49.9)	39.2 (32.6–47.2)	− 0.165 − 1.939	0.869 0.053	1021	1020
Binary**	2020/32–33	71.3 (55.5–91.7)	62.5 (47.9–81.6)	− 0.709 1.088	0.478 0.277	2955	2544
Binary**	2020/38–39	31.5 (25.9–38.2)	35.8 (25.9–39.0)	0.076 0.547	0.940 0.585	985	756

^*^No-choice tests were conducted in a climate chamber under ambient (400 ppm) or strongly elevated (750 ppm) CO_2_ concentrations. **Binary-choice tests were conducted with plants developed under ambient (400 ppm) or elevated (450 ppm) CO_2_ concentrations in the FACE system

## Discussion

Growing under elevated CO_2_ concentration altered the amount of single VOCs released from pear trees. Many herbivorous insects use specific blends of plant-emitted volatiles to identify and locate suitable host plants (Bruce and Pickett 2011). Therefore, the change in relative release of specific compounds in plant odors was expected to impact the behavior of the highly specialized pest insect *C. pyri*. Especially, terpenes and aldehydes are known to impact the behavior of different insect species. Thus, among other aldehydes, hexanal is shown to be perceived by *Cacopsylla pruni*, a psyllid species closely related to *C. pyri* (Gallinger et al. 2020). Contrary to expectations, the relative decrease of hexanal in the odor profile of pear trees had no impact on the host preference of *C. pyri* females. Neither had the relative increase of γ-terpinene. So far, it is unknown which compounds *C. pyri* can perceive (Gross et al. 2022), but in previous studies with C*acopsylla picta*, there was evidence that young individuals are attracted by the terpene β-caryophyllene (Mayer et al. 2008a, b). Until now, the role of specific aldehydes and terpenes for host finding and choice of *C. pyri* remains unknown. The olfactometer experiments revealed no significant attraction of *C. pyri* females to pear odors, indicating that host selection of *C.* *pyri* may not be based on plant volatiles alone. Previous studies on other psyllid species highlighted the importance of visual cues on host selection (Farnier et al. 2018; George et al. 2020; Patt et al. 2011; Wenninger et al. 2009). Further research should be undertaken to investigate the role of visual cues and possible synergistic effects of olfactory and visual cues for host detection of *C. pyri*.

Nonetheless, changes in VOC profiles could impact other pest insects such as moths or aphids. Due to the complexity and specificity of interactions, no general predictions can be made at this point. Different effects of atmospheric CO_2_ were found concerning other pest insects and host plants. For the corn leaf aphid *Rhopalosiphum maidis*, it is shown that winged and wingless adults preferred the volatile profile of barley (*Hordeum vulgare*) seedlings grown under ambient compared to elevated CO_2_ concentrations in two choice olfactometer trials (Chen et al. 2019). In contrast, soybean aphid *Aphis glycines* do not discriminate odors from undamaged soybean grown under ambient or elevated CO_2_ concentrations (O’Neill et al. 2010). Predators and parasitoids likewise rely on olfactory cues to find their prey. Anthocorid bugs, the most significant predators of *C. pyri*, are attracted to α-farnesene and methyl salicylate (Scutareanu et al. 2003). These plant volatiles are induced by psyllid feeding (Scutareanu et al. 1997, 2003). We did not find an impact of CO_2_ on the release of these compounds in our current study. Nevertheless, the effect of climate change parameters on herbivore-induced plant VOCs in the pear-psyllid-predator system is an important issue for future research.

Additionally, we found a variable influence of CO_2_ on pear VOCs in the two years of our studies. These differences are likely based on interactions with further climatic conditions. Especially, heat and water stress should be considered. Indeed, a high supply with CO_2_ can lead to an increase in photosynthesis rates, whereas high temperatures can impair the activity of photosystem II and reduce the photosynthetic efficiency (Havaux et al. 1991). The photosynthetic capacity of apple leaves was significantly reduced during daytimes with high temperatures and light intensities (Mihaljević et al. 2020). Additionally, the pear trees were placed in the middle of the FACE rings in 2020, while they were placed along the edges in 2019. The CO_2_ distribution inside the rings is most homogeneous in the middle of the ring system (data not shown). The CO_2_ concentration may therefore have been more variable in 2019 than in 2020. Another aspect that should be taken in to consideration is the duration of exposure to elevated CO_2_ concentrations. In this study, pear trees were exposed to higher CO_2_ levels for a certain period. We choose to expose the trees before bud break and investigate the plant VOCs and psyllid preferences during leaf and shoot development under CO_2_ exposition. As this is a critical time in the development of plant as well as psyllid populations, due to limitations in time and space, we were not able to cultivate pear trees for several years. Therefore, we cannot exclude further changes or adaptations of VOC release from pear trees after CO_2_ exposition for longer time periods.

Changes in volatile emissions from pear trees may indicate changes in plant metabolism. Impacts on primary metabolites are likely to affect feeding behavior, reproduction success, and survival of psyllids (Gallinger and Gross 2018, 2020). A recent study with *C. picta* demonstrated a correlation of C:N ratio in the host phloem sap and a oviposition preference of *C. picta* females, highlighting the impact of phloem sap composition for psyllid oviposition choice (Görg et al. 2020). Contrary to our expectations, the results from the oviposition experiments in the current study are not indicating a biologically relevant change in phloem sap quality due to the exposition of the trees to elevated CO_2_ levels.

Another important aspect in the investigated system is the transmission of phytopathogens by *C. pyri*. Bosquee et al. (2018) showed a higher transmission efficiency of Potato virus Y for *Myzus persicae* reared under elevated CO_2_ compared to aphids reared under ambient CO_2_ concentrations. Thus, the impact on feeding behavior and phytoplasma transmission efficiency of psyllids should be further investigated with regard on the spread of “*Ca*. P. pyri”, a phytopathogen that is vectored by *C. pyri*.

Additionally, CO_2_ concentrations can affect psyllid fitness and development. A positive impact on insect performance under elevated CO_2_ concentration was demonstrated for *A. glycines* on soybeans, by increased numbers of aphids (Dermody et al. 2008). In accordance, Guo et al. (2014) found increased growth rates of pea aphids (*Acyrthosiphon pisum*) on *Medicago truncatula* grown under increased CO_2_ concentration. These effects were attributed to differences in feeding behavior of aphids, due to decreased plant resistance (Guo et al. 2014). This again highlights the importance of further investigations on the interaction between psyllid vectors, phytoplasmas, and their host plants under future climate conditions.

## Supplementary Information

Below is the link to the electronic supplementary material.

Supplementary file1 (DOCX 17.6 KB)

## Data Availability

Not applicable.
