# Supplementary material for: Altered volatile emission of pear trees under elevated atmospheric CO2 levels has no relevance to pear psyllid host choice
Source: Environ Sci Pollut Res Int. 2023 Jan 20;30(15):43740–51. doi: 10.1007/s11356-023-25260-w (PMC10076355; doi:10.1007/s11356-023-25260-w)
Supplement: Supplementary file 1 — Supplementary file1 (DOCX 17.6 KB) [file 11356_2023_25260_MOESM1_ESM.docx]

**Altered volatile emission of pear trees under elevated atmospheric CO_2_ levels has no relevance to pear psyllid host choice**

Jannicke Gallinger^1,2*^, Margit Rid^1^, Annette Reineke^3^, Jürgen Gross^1^

^1^Institute for Plant Protection in Fruit Crops and Viticulture, Julius Kühn-Institut, Federal Research Institute for Cultivated Plants, Schwabenheimer Str. 101, D-69221 Dossenheim, Germany

^2^Department of Ecology, Swedish University of Agricultural Sciences, Ulls väg 16, 75007 Uppsala, Sweden

^3^Geisenheim University, Department of Crop Protection, Von-Lade-Str. 1, D-65366, Geisenheim, Germany

* Corresponding author: Jannicke Gallinger: [Jannicke.gallinger@slu.se](mailto:Jannicke.gallinger@slu.se);

Table S1: Detected compounds in headspace analysis of *Pyrus communis* trees and Retention indices (RI) used for identification in combination with mass spectra from authentic standards.

| Compound | RI |
| --- | --- |
| 1-Octene | 794 |
| Hexanal | 802 |
| Butyl acetate | 812 |
| (E)-2-hexen-1-al | 854 |
| Ethylbenzene | 857 |
| (Z)-3-hexen-1-ol | 861 |
| Xylene | 863/876 |
| 1-Nonene | 892 |
| Nonane | 900 |
| Heptanal | 904 |
| α-Pinene | 934 |
| (E)-2-heptenal | 959 |
| Benzaldehyde | 962 |
| β-Pinene | 978 |
| 6-Methyl-5-hepten-2-one | 987 |
| 1,3,5-Trimethylbenzene | 996 |
| Pseudocumene | 996 |
| 2-Carene | 999 |
| Decane | 1000 |
| Octanal | 1006 |
| (Z)-3-hexenyl-acetate | 1007 |
| α-Phellandren | 1008 |
| Hexyl acetate | 1012 |
| Limonene | 1018 /1031 |
| Cymene | 1026 |
| Ocimene | 1039/1049 |
| γ-Terpinene | 1060 |
| (E)-2-octenal | 1060 |
| Acetophenone | 1065 |
| RI.1071 | 1071 |
| Linalool oxide | 1089 |
| DMNT | 1094/1115 |
| RI.1098 | 1098 |
| Linalool | 1102 |
| Pelargonaldehyd | 1108 |
| Allo-Ocimene | 1129 |
| (E)-2-nonenal | 1163 |
| Ethylbenzoate | 1169 |
| Octanoic acid | 1182 |
| (Z)-3-hexenyl-butyrat | 1187 |
| Methyl salicylate | 1194 |
| Compound | RI |
| RI.1194 | 1194 |
| n-Dodecan | 1200 |
| Decanal | 1208 |
| (Z)-3-hexenyl-2-methyl-butanoat | 1238 |
| RI.1291 | 1291 |
| Tridecane | 1300 |
| Undecanal | 1308 |
| RI.1344 | 1344 |
| α-Copaene* | 1374 |
| β-Bourbonene* | 1385 |
| 1-Tetradecene | 1393 |
| Decanoic acid, ethyl ester | 1395 |
| Tetradecane | 1400 |
| Dodecanal | 1407 |
| β-Caryophyllene | 1423 |
| Geranyl acetone | 1450 |
| α-Humulene | 1460 |
| RI.1460 | 1460 |
| Alloaromadendrene | 1464 |
| RI.1470 | 1470 |
| RI.1487 | 1487 |
| RI.1496 | 1496 |
| Pentadecane | 1500 |
| RI.1505 | 1505 |
| α-Farnesene* | 1507 |
| RI.1528 | 1528 |
| (Z)-3-hexenyl-benzoate | 1568 |
| 1-Hexadecene | 1593 |
| Hexadecane | 1600 |
| RI.1670 | 1670 |
| RI.1695 | 1695 |
| Heptadecane | 1700 |
| Octadecane | 1800 |
| Nonadecane | 1900 |

* Compounds identified using the NIST Mass Spectral Search Program and comparison of RI from NIST Chemical WebBook
